# Associations of reproductive coercion and intimate partner violence with overt and covert family planning use among married adolescent girls in Niger

**DOI:** 10.1016/j.eclinm.2020.100359

**Published:** 2020-05-03

**Authors:** Jay G. Silverman, Sneha Challa, Sabrina C. Boyce, Sarah Averbach, Anita Raj

**Affiliations:** Center on Gender Equity and Health, University of California San Diego School of Medicine, 9500 Gilman Dr. La Jolla, San Diego, CA 92093, USA

**Keywords:** Intimate partner violence, Reproductive coercion, Family planning, Adolescent health, Early marriage, West Africa

## Abstract

**Background:**

In Niger the prevalence of girl child marriage and low female control over family planning (FP) has resulted in the world's highest adolescent fertility. Male control of FP is associated with intimate partner violence (IPV) and reproductive coercion (RC). We assessed associations of IPV and RC with FP use among married adolescent girls (ages 13–19 years) in Dosso, Niger (*N* = 1072).

**Methods:**

Multivariable, cross-sectional regression models assessed associations between physical IPV, sexual IPV, and RC and any FP use, FP use with husband knowledge (overt use), and FP use without husband knowledge (covert use).

**Findings:**

One in four married adolescent girls using FP reported doing so without husband's knowledge. Unadjusted and adjusted models indicated that physical IPV and RC were associated with covert FP use (vs. no use and vs. overt use), but not with overt use vs. no use. Only physical IPV remained significantly associated with covert use in models including all three forms of violence (AOR: 1.94 vs. any use; AOR: 3.63 vs. overt use).

**Interpretation:**

Married adolescents experiencing physical IPV or RC were more likely that others to use FP without their husbands’ knowledge. No form of GBV affected odds of FP use with husbands’ knowledge. Current results suggest caution regarding promoting engagement of men in decisions to use FP in this context, as this may undermine the reproductive autonomy of girls and women who will choose to use FP without the knowledge of their male partners.

**Research in Context**

**Evidence before this study**

Recent research in multiple global contexts has shown that male partners and/or other family members often engage in behaviors that actively and intentionally block girls’ and women's access to and use of FP methods, directly constraining reproductive autonomy. Intimate partner violence (IPV) is considered to be a key barrier to female reproductive autonomy. Intimate partner violence has been consistently found to be associated with childbearing at younger ages, high parity, and unintended pregnancy. Reproductive coercion (RC) is a form of gender-based violence that includes male partner or family behaviors that block women's access to or use of FP, or otherwise coerce women to become pregnant against their will. Studies have found that RC is associated but not collinear with IPV, and may represent a more proximal and direct link with FP use than IPV. A key coping strategy reported in multiple contexts by those facing RC is use of an FP method without the knowledge of a male partner, allowing such women and girls to retain their reproductive autonomy in the face of RC.

**Added value of this study**

Little research has been conducted on RC in low or middle-income countries, and no studies of RC have been conducted with married adolescents. Further, among the small body of studies assessing associations of RC and FP use, no research has examined whether these associations differ based on whether this use is with or without male partner knowledge (i.e., overt vs. covert FP use). Given that constraints on married adolescents’ reproductive autonomy are considered a key barrier to their use of FP and their broader health and development in Niger and elsewhere, the current study extends the existing state of knowledge by assessing associations of IPV and RC with FP use, both overt and covert, among a population-based sample of married adolescent girls residing in three large districts of the Dosso region of Niger.

**Implications of all available evidence**

The findings that a large portion of married adolescents in this context who use FP methods are choosing to do so without the knowledge of or participation from their husbands, and that this is often in the context of physical partner violence (28%) and reproductive coercion (25%) from these male partners, may have important implications for the increasing number of programs and policies that seek to increase utilization of FP via male involvement in FP decisions.

Alt-text: Unlabelled box

## 1 Introduction

Health outcomes for adolescent girls in Niger have remained persistently poor despite increased focus on this vulnerable population within international development investments, programming and policies [[1], [2]–3]. This has been attributed, in part, to extremely high rates of child marriage and both low use and of and lack of autonomy regarding family planning (FP) among married girls in this context [4]. In 2016, Niger had the highest rate of child marriage in the world, with 28% of girls marrying by the age of 15 years and 76% marrying by age 18 years [5]. Early marriage is known to be associated with both low FP use, high unmet need for FP, and intimate partner violence (IPV) [[6], [7], [8], [9]–10]. Early childbearing accompanies child marriage; with 187 births per 1000 girls and young women ages 15–19 years in 2017 [11], Niger has the highest adolescent fertility globally. The negative outcomes associated with early and inadequately spaced childbearing include maternal, infant, and under 5 years mortality, often related to low birth weight [[12], [13]–14]. The government of Niger has set a goal of 50% modern contraceptive prevalence rate (mCPR) as a signatory to FP2020, a global partnership for investment in rights-based family planning [15,16]. However, as of 2019, mCPR has reached only 15.5% (18.5% among married women) [17,18].

Significant barriers to FP method access and use are known to exist at multiple levels of adolescents’ social environment [[19], [20], [21]–22]. Research in multiple low- and middle-income countries has identified adolescent girls’ low autonomy as a critical barrier to modern FP method use [[23], [24], [25]–26]. Social norms in sub-Saharan Africa often dictate that male partners hold decision-making power regarding FP method use and choice [23,[27], [28], [29], [30]–31]. In fact, recent research in multiple global contexts has shown that male partners and/or other family members often engage in behaviors that actively and intentionally block girls’ and women's access to and use of FP methods, directly constraining reproductive autonomy [[32], [33], [34]–35].

Intimate partner violence (IPV) is considered to be a key barrier to female reproductive autonomy. Intimate partner violence has been consistently found to be associated with childbearing at younger ages, high parity, and unintended pregnancy [33,[36], [37]–38]. The lifetime prevalence of physical and/or sexual IPV is estimated to be greater than 1 in 3 in sub-Saharan Africa [39] and young women ages 15–24 years experiencing IPV have highest levels of unmet need for FP in the region [38]. Reproductive coercion (RC) is a form of gender-based violence that includes male partner or family behaviors that block women's access to or use of FP, or otherwise coerce women to become pregnant against their will [40]. Studies have found that RC is associated but not collinear with IPV, and may represent a more proximal and direct link with FP use than IPV. Recent trials of clinic-based interventions to address RC indicate that health care providers can successfully assist women and girls reduce these experiences [41,42]. A key coping strategy reported in multiple contexts by those facing RC is use of an FP method without the knowledge of a male partner, allowing such women and girls to retain their reproductive autonomy in the face of RC [[43], [44]–45].

Little research has been conducted on RC in low or middle-income countries, and no studies of RC have been conducted with married adolescents. Further, among the small body of studies assessing associations of RC and FP use, no research has examined whether these associations differ based on whether this use is with or without male partner knowledge (i.e., overt vs. covert FP use) [46]. Given that constraints on married adolescents’ reproductive autonomy are considered a key barrier to their use of FP and their broader health and development in Niger and elsewhere [[47], [48], [49]–50], the current study seeks to extend the existing state of knowledge by assessing associations of IPV and RC with FP use, both overt and covert, among a population-based sample of married adolescent girls residing in three large districts of the Dosso region of Niger.

## 2 Methods

### 2.1 Data source

Cross-sectional data used for this analysis were collected from April-June 2016 as part of the baseline survey for the *Reaching Married Adolescents (RMA) Study* [51]. This ongoing study employs a four-arm cluster randomised control design to evaluate the effects of multiple community-based approaches to expand access to modern FP methods among married adolescents in the Dosso region of Niger. A two-stage random sampling approach was used whereby the first stage involved random selection of 16 villages, across each of three districts, for inclusion based on the following criteria: (1) located in the Dosso, Doutchi, or Loga districts of the Dosso region, (2) having at least 1000 inhabitants, and (3) Hausa- or Zarma-speaking. In each district, from the 16 randomly selected villages 25 households were randomly selected for participation from each village based on a listing of married adolescent girls provided by village chiefs. Girls were considered eligible if they were: (1) between 13–19 years of age, (2) married, (3) fluent in Hausa or Zarma, (4) not having plans to move away in the next 18 months or plans to travel for more than 6 months during that time, (5) not currently sterilized, and (6) willing and able to provide informed consent. Further details on sampling for this study and allocation to intervention or control are not relevant to the present analysis but can be found in the paper describing the study protocol ([51] – https://www.ncbi.nlm.nih.gov/pmc/articles/PMC6921454/). Based on low levels of written literacy, verbal informed consent was obtained prior to survey participation. Precautions aligned with the World Health Organization guidelines for ethical conduct of research on violence against women [52] were taken to ensure the safety of participants, their privacy, and the confidentiality of their responses. Our study was approved by both the University of California San Diego School of Medicine Institutional Review Board and the Research Ethics Board of the Niger Ministry of Health.

Research Assistants (RAs) who were trained, gender-matched, and fluent in French and Hausa and/or Zarma collected self-report data from participants using quantitative survey interview methods. The selected households were first approached by RAs to introduce the study and confirm the presence of an eligible married adolescent girl. Permission from male heads of household was sought prior to married adolescent girls being approached in order to comply with local customs; no cases of non-participation related to denial of such permission. Up to three visits were made to each household, after which no additional visits were made. If a household approached either did not respond or did not have an eligible married adolescent girl, another household in the village was randomly selected from those not selected during the initial round as a replacement. Interviews were conducted using pre-programmed tablet devices in a private location selected by participants; interviews required 40–60 min to complete. In total, data were collected from 1072 participants. Of all the married adolescent girls selected for recruitment, 88.0% participated in the baseline survey.

### 2.2 Data statement

Due to the sensitivity of the data used for this analysis, additional information including deidentified participant data and a data dictionary, can be made available upon reasonable request. Please contact the UC San Diego Center on Gender Equity and Health (GEH@ucsd.edu) for any data requests.

### 2.3 Measures

Ever using a reversible modern FP method among this young sample – any, with husband's knowledge (overt use), and without a husband's knowledge (covert use) – were the primary outcomes of this analysis. Participants were asked if they had ever done anything or used any method to space or delay pregnancy. If they responded “yes”, and stated that they currently used or in the past had used a modern method including: (1) intrauterine devices (IUDs), (2) injectable contraceptives, (3) contraceptive implants, (4) oral contraceptive pills, (6) male condoms, (7) female condoms, (8) emergency contraception, or (9) lactation amenorrhea method, they were considered to have ever used any modern FP method. Participants were also asked if their husbands knew that they had ever done something, or used an FP method, to space or delay pregnancy. Family planning method use with or without husbands’ knowledge was coded use the following categories: (1) no use, 92) covert use, (3) overt use.

Physical and sexual IPV and RC were studied as the main exposures of interest. The measure of RC consisted of six items regarding experiences of male partners’ interference with FP method use, including pregnancy pressure and contraceptive sabotage from a measure of RC previously validated in the U.S. [53] that was adapted to the Niger context based on formative data and stakeholder input. Participants were asked whether their husbands had ever either 1() tried to force or pressure them to become pregnant, (2) taken their FP method away from them, (3) kept them from going to the clinic to access FP methods, (4) said they would leave if they did not get pregnant, or (5) hurt them physically because they did not get pregnant. The sixth item asked if anyone including their husbands, in-laws, or co-wives pressured them, made them feel badly, or treated them badly in the past 12 months for not having a child (standardized Cronbach's alpha=0•72 for these six items). Those that responded positively to any of these six items were considered as having ever experienced RC.

The physical IPV and sexual IPV measures included items adapted from the Demographic and Health Survey (DHS) domestic violence module based on the WHO multi-country study [54]. These were also dichotomous variables with participants considered as having experienced physical IPV if they reported via six items that their husbands had ever pushed them, slapped them, twisted their arms or pulled their hair, hit them with their fists or something that could hurt them, kicked/dragged/beaten them up, or tried to choke/burn them. Participants were considered as having experienced sexual IPV if they reported that their husbands had ever either physically forced them to have sexual intercourse when they did not what to or if their husbands had ever physically forced them to perform any other sexual acts they did not want to. For all exposure and outcome measures, only those that provided valid responses for all relevant items were included in analysis. If participants had missing data or responded ‘other’, ‘don't know’, or ‘decline to answer’, they were considered missing for that variable. Missing data did not exceed 10% for any exposure or outcome variables.

Potential covariates included in these analyses were: participant age, age difference between husband and participant, participant age at marriage, participant education, husband education, food insecurity (as a proxy for economic insecurity), number of co-wives, parity (number of live biological children), whether the husband had migrated away from the village for three months or more during the past year, and district. This demographic information was collected from heads of household during the Household Recruitment Survey. Wife's age, age difference between husband and wife, and wife's age at marriage were all treated continuously. Education level for both wives and husbands was captured by a survey item asking if each person had ever attended government (i.e., government funded – providing education in topics including reading, writing, math, etc.) or Quranic (sponsored and provided by religious organizations) school to which responses were, “yes, (s)he attended government school,” “yes (s)he attended Quranic school,” “no schooling but can read or write,” and “no (s)he did not attend school and does not read or write.” For analysis, exclusive categories were created corresponding to government school, Quranic school and no government school, and no education (comprising “no schooling but can read or write,” and “no (s)he did not attend school and does not read or write”). Since men may have had children with more than one wife, the adolescent wife participant's number of children was used to represent the couple's parity (number of living children). This was categorized as no children, one child, or two or more children. The number of wives each male participant had was determined and categorized to represent those in monogamous unions (one wife) or polygamous unions (more than 1 wife). Food insecurity, a measure of wealth or economic security, was a dichotomous variable represented by an item that asked whether in the month prior to the interview the participant or any member of their family had gone without eating the whole day because there was not enough food. Finally, given the realities of migration in this population, a dichotomous variable was created to represent whether husbands had spent more than 3 months away from the village in the past year.

### 2.4 Statistical analysis

Analyses presented here were conducted using *SAS Studio*® (SAS Institute Inc., 2018)*.* First, descriptive statistics for demographic characteristics were examined by FP use outcome variables using t-tests, ANOVAs, and chi-squared tests to examine differences by group [Table 1]. Modeling followed a backwards stepwise approach. Unadjusted logistic regression models including each individual exposure (physical IPV, sexual IPV, and RC) and each individual outcome (ever use of modern FP and multinomial overt/covert/no modern FP method) were run [Tables 2 and 3] to determine unadjusted odds ratios (ORs) and 95% confidence intervals (CI). After this, logistic regression models were constructed that included covariates found to be significant at the *p*<0.20 level in the t-tests, ANOVAs, or chi-squared tests to determine adjusted odds ratios (AORs) and 95% CIs. These models were then reduced, keeping only those covariates significant at the *p*<0.10 level. Finally, models were constructed to include all three forms of violence in addition to any of the covariates significant at the *p*<0.10 level in the reduced models.

**Table 1:** Sample demographics by outcomes.

	Total	Ever use of modern FP		Covert, overt modern FP use			
		Yes		No use	Overt	Covert	
		(*N* = 130)		(*N* = 925)	(*N* = 98 )	(*N* = 32)	
Variables	*N* (%)	*N* (%)	*p*-value	*N* (%)	*p*-value	*N* (%)	*p*-value
**Wife's age**							
13–14 years	49 (4.57)	2 (1.54)	<0.001*	45 (4.86)	1 (1.02)	1 (3.13)	<0.001⁎⁎
15–17 years	452 (42.16)	34 (26.15)		412 (44.54)	28 (28.57)	6 (18.75)	
18–19 years	571 (53.26)	94 (72.31)		468 (50.59)	69 (70.41)	25 (78.13)	
**Husband's age**							
15–24 years	491 (45.80)	43 (33.08)	<0.001*	442 (47.78)	31 (31.63)	12 (37.50)	<0.001⁎⁎
25–29 years	327 (30.50)	47 (36.15)		271 (29.30)	35 (35.71)	12 (37.50)	
30 years and over	222 (20.71)	37 (23.85)		183 (19.78)	30 (30.61)	7 (21.88)	
**Age difference**							
0–4 years	205 (19.12)	21 (16.15)	0.014*	182 (19.68)	13 (13.27)	8 (25.00)	0.002⁎⁎
5–6 years	257 (23.97)	24 (18.46)		230 (24.86)	19 (19.39)	5 (15.63)	
7–9 years	260 (24.25)	31 (23.85)		224 (24.22)	24 (24.49)	7 (21.88)	
10 years and over	318 (29.66)	51 (39.23)		260 (28.11)	40 (40.82)	11 (34.38)	
**Wife's age at marriage**							
7–13 years	391 (36.47)	63 (48.46)	<0.001*	320 (34.59)	51 (52.04)	12 (37.50)	<0.001⁎⁎
14–15 years	428 (39.93)	46 (35.38)		376 (40.65)	36 (36.73)	10 (31.25)	
16–17 years	213 (19.87)	20 (15.38)		190 (20.54)	11 (11.22)	9 (28.13)	
18–19 years	37 (3.45)	0 (0.00)		37 (4.00)	0 (0.00)	0 (0.00)	
**Wife's parity**							
None	429 (40.02)	7 (5.38)	<0.001^	414 (44.76)	1 (1.02)	6 (18.75)	<0.001^
1 birth	356 (33.21)	48 (36.92)		302 (32.65)	34 (34.69)	14 (43.75)	
2 or more births	287 (26.77)	75 (57.69)		209 (22.59)	63 (64.29)	12 (37.50)	
**Wife's education**							
*Government School*	372 (34.70)	45 (34.62)	0.073^	319 (34.49)	36 (36.73)	9 (28.13)	0.082^a^
*Quranic School*	175 (16.32)	29 (22.31)		142 (15.35)	24 (24.49)	5 (15.63)	
*No Education*	516 (48.13)	53 (40.77)		458 (49.51)	36 (36.73)	17 (53.13)	
**Husband's education**							
*Government School*	502 (46.83)	60 (46.15)	0.002^	432 (46.70)	43 (43.88)	17 (53.13)	0.007^a^
*Quranic School*	215 (20.06)	40 (30.77)		173 (18.70)	33 (33.67)	7 (21.88)	
*No Education*	318 (29.66)	27 (20.77)		286 (30.92)	20 (20.41)	7 (21.8)	
**Number of wives**							
*Monogamous*	897 (83.68)	112 (86.15)	0.49^	770 (83.24)	85 (86.73)	27 (84.38)	0.77^a^
*Polygamous*	143 (13.34	15 (11.54)		126 (13.62)	11 (11.22)	4 (12.50)	
**Food insecurity**							
*No*	835 (77.89)	95 (73.08)	0.15^	725 (78.38)	71 (72.45)	24 (75.00)	0.34^a^
*Yes*	234 (21.83)	35 (26.92)		197 (21.30)	27 (27.55)	8 (25.00)	
**Has husband spend >3 months away**							
*No*	313 (29.20)	38 (29.3)	0.99^	267 (28.86)	29 (29.59)	9 (28.13)	0.99⁎⁎
*Yes*	722 (67.35)	89 (68.46)		624 (67.46)	67 (68.37)	22 (68.75)	
**Exposures**							
**Physical IPV**							
*No*	976 (91.04)	109 (83.85)	0.002^	853 (92.22)	87 (88.78)	22 (68.75)	<0.001⁎⁎
*Yes*	88 (8.21)	20 (15.38)		68 (7.35)	11 (11.22)	9 (28.13)	
**Sexual IPV**							
*No*	995 (92.82)	122 (93.85)	0.98^	861 (93.08)	93 (94.90)	20 (90.63)	0.53⁎⁎
*Yes*	57 (5.32)	7 (5.38)		50 (5.41)	4 (4.08)	3 (9.38)	
**RC**							
*No*	883 (82.37)	109 (83.85)	0.066^	765 (82.70)	85 (86.73)	24 (75.00)	0.025⁎⁎
*Yes*	109 (10.17)	20 (15.38)		86 (9.30)	12 (12.24)	8 (25.00)	

Abbreviations: FP—Family Planning, IPV—Intimate Partner Violence, RC—Reproductive Coercion.

⁎ Results from t-tests.

⁎⁎ Results from ANOVA.

^ Results from Chi-square tests. ^^^^Results from Fisher's Exact test.

**Table 2:** Modeling use of a modern family planning method (inclusive of covert and overt use).

	Ever modern family planning use		
	Crude	Adjusted models†	Final models††
	OR	AOR	AOR
	(95% CI)	(95% CI)	(95% CI)
	*p*-value	*p*-value	*p*-value
**Ever experienced husband physical IPV**			
*Yes*	**2.3**	**2.06**	**1.94**
	**(1.35, 3.94)**	**(1.13, 3.73)**	**(1.04, 3.64)**
	***0*.*002***	***0*.*018***	***0*.*039***
**Ever experienced husband sexual IPV**			
*Yes*	0.99	1.04	0.71
	(0.44, 2.23)	(0.43, 2.54)	(0.28, 1.81)
	*0.98*	*0.93*	*0.47*
**Ever experienced reproductive coercion**			
*Yes*	**1.63**	1.34*	1.26**
	**(0.96, 2.76)**	(0.73, 2.45)	(0.69, 2.32)
	***0*.*068***	*0.35*	*0.45*

Abbreviations: FP—Family Planning, IPV—Intimate Partner Violence, RC—Reproductive Coercion.

† Included covariates parity, district; parity and district significant at *p*<0.05.

†† Included covariates parity, district, all forms of violence; parity and district significant at *p*<0.05.

⁎ Included covariates parity, food insecurity, district; parity and district significant at *p*<0.05.

⁎⁎ Included covariates parity, food insecurity, district, all forms of violence; parity and district significant at *p*<0.05.

**Table 3:** Modeling multinomial covert, overt modern family planning method use.

Covert, overt modern family planning use									
	Overt vs no use			Covert vs no use			Covert vs overt use		
	Crude (Overt vs No Use	Adjusted (Overt vs No Use)†	Final (Overt vs No Use)††	Crude (Covert vs No Use)	Adjusted (Covert vs No Use)†	Final (Covert vs No Use)††	Crude (Covert vs Overt)	Adjusted (Covert vs Overt)†	Final (Covert vs Overt)††
	OR	AOR	AOR	OR	AOR	AOR	OR	AOR	AOR
	(95% CI)	(95% CI)	(95% CI)	(95% CI)	(95% CI)	(95% CI)	(95% CI)	(95% CI)	(95% CI)
	*p*-value	*p*-value	*p*-value	*p*-value	*p*-value	*p*-value	*p*-value	*p*-value	*p*-value
**Physical IPV**									
*Yes*	1.59	1.23	1.23	**5.13**	**5.55**	**4.48**	**3.24**	**4.51**	**3.63**
	(0.81, 3.11)	(0.59, 2.57)	(0.57, 2.67)	**(2.27, 11.58)**	**(2.36, 13.04)**	**(1.74, 11.51)**	**(1.19, 8.77)**	**(1.59, 12.81)**	**(1.17, 11.29)**
	*0.18*	*0.58*	*0.59*	***<0*.*001***	***<0*.*001***	***0*.*002***	***0*.*021***	***0*.*005***	***0*.*026***
**Sexual IPV**									
*Yes*	0.74	0.69	0.59	1.78	2.29	1.00	2.41	**4.47**	1.70
	(0.26, 2.10)	(0.23, 2.12)	(0.19, 1.88)	(0.53, 6.05)	(0.64, 8.17)	(0.25, 3.96)	(0.51, 11.38)	**(1.57, 12.70)**	(0.31, 930)
	*0.57*	*0.52*	*0.37*	*0.35*	*0.2*	*0.99*	*0.27*	**0.005**	*0.54*
**RC**									
*Yes*	1.26	0.83	0.97	**2.97**	**3.41**	2.02	2.36	**4.1**	2.09
	(0.66, 2.39)	(0.39, 1.77)	(0.47, 2.00)	**(1.29, 6.80)**	**(1.39, 8.34)**	(0.76, 5.39)	(0.87, 6.44)	**(1.36, 12.35)**	(0.66, 6.57)
	*0.49*	0.63	*0.94*	***0*.*01***	***0*.*007***	*0.16*	*0.093*	***0*.*012***	*0.21*†, ††

Abbreviations: FP—Family Planning, IPV—Intimate Partner Violence, RC—Reproductive Coercion.

† Included covariates parity, district; parity and district significant at *p*<0.05.

†† Included covariates parity, district, all forms of violence; parity and district significant at *p*<0.05.

### 2.5 Role of the funding source

This work was supported by the 10.13039/100000865Bill and Melinda Gates Foundation (OPP1195210, Prime: Pathfinder International; Research PI: J Silverman). The funder had no role in the study design, the collection/analysis/interpretation of data, the writing of this manuscript, or the decision to submit it for publication. Dr. Silverman has full access to all data related to this study. All the authors read and approved the final manuscript.

## 3 Results

### 3.1 Demographics

The final sample for these analyses included 1072 married adolescent girls between the ages of 13–19 years. The sample was skewed toward older adolescents, with the majority (53.3%) over the age of 17 years [Table 1]. Almost all (96.3%) were married before age 18 years, with 39.9% married between the ages of 14–15. There were large age differences found between adolescent wives and their husbands, with 29.7% of men at least 10 years older than their adolescent wives. Lack of education was prevalent among both adolescent wives and their husbands, with almost half of adolescent wives (48.1%) reporting no education and 29.7% of the husbands reporting no education. Two in five participating couples had no children. All covariates listed in Table 1 were associated with one or more of the assessed outcomes at the *p*<0.05 level, with the exception of the number of co-wives and husband's migration status. Our exposures of interest (lifetime physical IPV, sexual IPV, and RC) were found to have a prevalence of 8.2%, 5.3%, and 10.2%, respectively. Approximately 1 in 8 (12.1%) married adolescent girls in our sample reported ever using a modern FP method, 3·0% (24·6% of all FP users) reported having ever used an FP method without their husband's knowledge.

### 3.2 Associations of IPV and RC with FP outcomes

For the outcome of any modern FP method use [Table 2], physical IPV was found to be significantly associated in the unadjusted model (OR: 2.30, 95% CI: 1.35, 3.94), the multivariable model adjusting for covariates significant at the *p*<0.10 level (OR: 2.06, 95% CI: 1.13, 3.73), and the final multivariable models adjusting for significant covariates and the other two forms of violence – RC and sexual IPV (OR: 1.94, 95% CI: 1.04, 3.64). Reproductive coercion was found to be associated with ever use of modern FP methods in the unadjusted model, but only as a nonsignificant trend (OR: 1.63, 95% CI: 0.96, 2.76; *p* = 0.068) and no significant associations were present in either multivariate model. In all adjusted models, covariates parity and district were retained with both reaching significance at *p*<0.05. In models with RC as the exposure, food insecurity was also retained based on reaching significance at *p*<0.1 but did not reach significant at *p*<0.05. No associations were found between sexual IPV and any FP outcomes.

When FP use was categorized based on whether it was without or with husbands’ knowledge (i.e., covert vs. overt use) and compared with no use via multinomial models [Table 3], no form of violence was found to be predictive of overt use. However, when covert use was compared to no use, physical IPV was associated with higher odds of covert use compared to non-use in unadjusted models (OR: 5.13, 95% CI: 2.27, 11.50), after adjusting for covariates (AOR: 5.55, 95% CI: 2.6, 13.04), and also after controlling for the other two forms of violence in addition to covariates (AOR: 4.48, 95% CI: 1.74, 11.51). RC was also found to be associated with covert use compared to non-use in the unadjusted model (OR: 2.97, 95% CI: 1.29, 6.80) and after adjusting for covariates (AOR: 3.41, 95% CI: 1.39, 8.34), but did not retain significance in models inclusive of physical and sexual IPV (*p* = 0.16). Covariates parity and district were retained in all adjusted models with both, both of which were significant at *p*<0.05. No significant associations were found between sexual IPV and FP use in this or any other multinomial model.

Similarly, when comparing covert FP use to overt use in multinomial models, associations were found between physical IPV and covert use (vs. overt use) in unadjusted models (OR: 3.24, 95% CI: 1.19, 8.7), after controlling for covariates (AOR: 4.51, 95% CI: 1.59, 12.81), and also after including the other two forms of violence alongside covariates (AOR: 3.63, 95% CI: 1.17, 11.29). RC was associated with covert use compared to overt use only at the level of a nonsignificant trend in the unadjusted model (*p* = 0.093) but did reach significance in the model adjusted for covariates (AOR: 4.10, 95% CI: 1.36, 12.35). However, RC did not remain significantly associated with covert vs. overt use in the model adjusted for both forms of IPV. Similarly, sexual IPV reached statistical significance as a predictor of covert vs. overt use only after inclusion of covariates (AOR: 4.47, 95% CI: 1.57, 12·70), but did not retain significance in the model adjusted for other forms of GBV. Again, in all adjusted models, parity and district were retained and reached significance at *p*<0.05.

## 4 Discussion

Among this population-based sample of married adolescent girls living in three rural districts of the Dosso region of Niger, approximately 1 in 10 reported reproductive coercion, i.e., having had their access to or use of FP reduced or having been coerced or forced to become pregnant against their will. Approximately 1 in 12 reported ever having experienced physical violence from an intimate partner, and 1 in 20 reported ever having experienced sexual violence. One in four married adolescents who reported ever having used a modern FP method, reported that they had done so without the knowledge of their husbands (i.e., used FP covertly). Among those experiencing physical IPV and RC, covert use of FP was significantly more likely. The finding that a large portion of married adolescents in this context who use FP methods are choosing to do so without the knowledge of or participation from their husbands, and that this is often in the context of physical violence (28%) and reproductive coercion (25%) from these male partners, may have important implications for the increasing number of programs and policies that seek to increase utilization of FP via male involvement in FP decisions [55,56].

Promoting norms of FP method acceptance and increasing knowledge of FP among men may be safe and effective approaches to reduce barriers to women's FP method use [[57], [58], [59]–60]. However, direct involvement of men in decisions regarding girls’ and women's use of an FP method (particularly, a female-controlled method) may undermine the reproductive autonomy of women who do not feel safe to include their partners. Thus, in order to ensure that FP programming and policies are rights-based and women-centered (as mandated by the WHO and FP2020) [61,62], male involvement in FP counseling and decisions should be based on a woman's active choice to involve that partner. While we cannot know from the current study the prevalence of choosing to use FP methods covertly, or whether this choice is associated with GBV, beyond the current context of adolescent wives in rural Niger, this finding is consistent with a small but growing body of literature documenting that approximately 1 in 4 women in Sub-Saharan countries who use contraception choose to do so covertly [63,64]. These findings highlight the critical need for research to understand women's and girls’ desires and choices regarding involvement of male partners in FP decision-making and, importantly, to explore the practical applications of these findings to health sector programs providing FP service to women and girls, as well programs to promote reproductive health via male partner engagement.

Supporting this recommendation, current findings indicate that married adolescents’ experiences of physical violence from male partners, as well as those of coercion by these men in order to thwart their FP method use, were linked to greater odds of these adolescents choosing to use FP methods covertly, i.e., without their husbands’ knowledge. In contrast, neither IPV nor RC were found to relate to adolescents’ use of FP with partner knowledge (i.e., overt use) when compared to no FP use. Because previous studies of gender-based violence and FP use have not assessed covert use vs. overt use, current findings cannot be directly compared to those from prior research, but they may advance our understanding of gender-based violence and how its associations with FP use may differ based on the specific nature of that use.

The current results may also help to explain the widespread inconsistency in the research literature, i.e., studies finding both positive and negative associations of IPV and FP use [[65], [66], [67], [68], [69], [70]–71]. Based on present findings and the patterns of findings across national contexts observed in earlier studies, including consideration of the FP methods mix available in these contexts, we posit that IPV and RC will consistently be *positively* associated with FP method use (particularly covert use) in contexts where female-controlled FP methods (e.g., IUDs, injectables, implants) are available and acceptable (i.e., widely used). However, where use of male-controlled FP methods (i.e., male condoms) is prevalent, we would expect that IPV and RC would be *negatively* associated with FP method use. More difficult to posit is the nature of the association of contraceptive pill use and IPV, as this will depend on whether the context allows for female control of this method, i.e., whether daily use of this method is possible without detection. If pills can easily be used without detection, we would expect this association to be positive; if it is difficult for a woman or girls to use pills without detection, then we would expect that IPV and RC would be negatively associated with using this method in this context.

These hypotheses are consistent with the extensive literature in the area of HIV risk that indicates that men who perpetrate IPV are less likely to use condoms with female partners [70]. Among the current sample of over 1000 married adolescents residing in rural Niger (where pill use can be considered as female-controlled due to husbands typically not co-residing with wives), less than 1% of participants reported that their husband used a male condom, with all of other modern methods used being female-controlled (i.e., pills, injectables, implants; IUD use was not reported in this context), a mix which we believe is responsible for the positive associations between IPV/RC and covert FP method use in the current study. In contrast, a recent study among a representative sample of women in Uttar Pradesh, India, where male condoms account for the majority (60%) of FP method use, found that both IPV and RC were *negatively* associated with FP use [72]. Finally, regardless of FP method mix, we would expect that IPV and RC will not be associated with *overt* FP method use, i.e., use involving male partner tacit or expressed consent.

Lastly, unlike earlier studies in the U.S. and the previously mentioned recent study in India [72,73], RC was not found to be associated with FP method use independently of IPV among married adolescent girls in Niger. Although AORs for RC in models inclusive of IPV comparing covert to no use and covert to overt use were greater than 2.0, there was inadequate power in the current study to conclude that these effects were statistically significant (*p*-values: 0.15 and 0.21, respectively). Future studies with great statistical power (e.g., larger Ns, higher prevalence of FP use and/or RC) are needed to consider whether RC is related to FP use in this context. Although underpowered, these findings regarding the associations between RC and covert use, as well as the association of RC and IPV, indicate convergent validity of the currently utilized measure of RC.

Such research may have important implications for practice. Screening for IPV is currently recommended in the context of FP counseling by multiple international bodies (e.g., WHO) [74]. Previous findings indicating that likelihood of FP method use is independently related to both IPV and RC support implementation of protocols that include screening for both IPV and RC. Future research in this vein may also inform whether inclusion of provider assessment of use or intention to use FP methods overtly vs. covertly, in combination with IPV/RC assessment, may facilitate counseling better tailored to a woman or girl's situation, needs and choices. A program integrating this combination of assessments within FP counseling has been found effective in multiple trials in the U.S. [41,42] Further epidemiologic and operational research inclusive of RC as a factor in women's and girls’ reproductive health is needed to improve the current state of knowledge and programming regarding FP method use.

The findings of the current study should be considered in the light of several design limitations. All analyses were cross-sectional, thus causality and temporality of associations cannot be assumed. Second, all data were provided via self-report of participants through face-to-face interviewing. Thus, there is potential for biases in reporting based on social desirability. Due to the restrictions for village and participant selection, the generalizability of the results are limited to the married adolescent girls in the Dosso region who matched these criteria. Despite this, the present findings represent an important advance given the limited understanding of the associations between experiences of GBV and FP use among such vulnerable populations. Lastly, the experiences reported may have occurred several years prior to their reporting, leading to potential for data being affected by recall bias. However, the potential for recall bias is likely reduced in the current study relative to other similar investigations due to the period of recall being limited by the young age of participants and related recency of marriage.

Our study provides evidence that experiences of gender-based violence, specifically physical IPV and RC, are associated with higher odds of modern FP method use among our sample of adolescent wives, but only when this use is without the knowledge of a male partner (i.e., covert). This is in contrast to negative associations of IPV and RC with FP use seen in other low resource settings where, unlike Niger, the majority of FP method use precludes female control of use [72]. While community-level male engagement to support social norms accepting of FP use are likely critical to increasing FP use in the longer term, approaches that support the direct involvement of men in women's decisions regarding FP use, either in community or clinic-based contexts, will not be safe or in the best interest of women or girls who have made the choice to use FP without the knowledge of their male partners. Thus, as discussed earlier, involvement of male partners in such programming should only take place after a woman or girl has indicated her interest in having her male partner involved in her FP decisions. The current findings indicate that programs implemented in contexts of prevalent adolescent marriage and childbearing (e.g., Niger), may wish to consider incorporating screening for both IPV and RC into FP counseling, as well as assessing desire to use FP covertly in order to inform FP method counseling and support women's and girls’ reproductive autonomy and health.

## Declaration of Competing Interest

Dr. Silverman reports grants from Bill and Melinda Gates Foundation during the conduct of the study.

Ms. Challa, Ms. Boyce, Dr. Averbach, and Dr. Raj have nothing to declare.
